# Evaluation of carbon nanotube probes in critical dimension atomic force microscopes

**DOI:** 10.1117/1.JMM.15.3.034005

**Published:** 2016-08-26

**Authors:** Jinho Choi, Byong Chon Park, Sang Jung Ahn, Dal-Hyun Kim, Joon Lyou, Ronald G. Dixson, Ndubuisi G. Orji, Joseph Fu, Theodore V. Vorburger

**Affiliations:** a Korea Research Institute of Standards and Science, 267 Gajeong-Ro, Yuseong-Gu, Daejeon 305-345, Republic of Korea; b Chungnam National University, 99 Daehak-Ro, Yuseong-Gu, Daejeon 34134, Republic of Korea; c National Institute of Standards and Technology, 100 Bureau Drive, Gaithersburg, Maryland 20899-8212, United States

**Keywords:** critical dimension atomic force microscope, atomic force microscope, carbon nanotube tip, nanomanipulation, ion beam bending, ion beam induced deposition, critical dimension metrology

## Abstract

The decreasing size of semiconductor features and the increasing structural complexity of advanced devices have placed continuously greater demands on manufacturing metrology, arising both from the measurement challenges of smaller feature sizes and the growing requirement to characterize structures in more than just a single critical dimension. For scanning electron microscopy, this has resulted in increasing sophistication of imaging models. For critical dimension atomic force microscopes (CD-AFMs), this has resulted in the need for smaller and more complex tips. Carbon nanotube (CNT) tips have thus been the focus of much interest and effort by a number of researchers. However, there have been significant issues surrounding both the manufacture and use of CNT tips. Specifically, the growth or attachment of CNTs to AFM cantilevers has been a challenge to the fabrication of CNT tips, and the flexibility and resultant bending artifacts have presented challenges to using CNT tips. The Korea Research Institute for Standards and Science (KRISS) has invested considerable effort in the controlled fabrication of CNT tips and is collaborating with the National Institute of Standards and Technology on the application of CNT tips for CD-AFM. Progress by KRISS on the precise control of CNT orientation, length, and end modification, using manipulation and focused ion beam processes, has allowed us to implement ball-capped CNT tips and bent CNT tips for CD-AFM. Using two different generations of CD-AFM instruments, we have evaluated these tip types by imaging a line/space grating and a programmed line edge roughness specimen. We concluded that these CNTs are capable of scanning the profiles of these structures, including re-entrant sidewalls, but there remain important challenges to address. These challenges include tighter control of tip geometry and careful optimization of scan parameters and algorithms for using CNT tips.

## 1 Introduction

The decreasing size of semiconductor features and the increasing structural complexity of advanced devices have placed continuously greater demands on manufacturing metrology, arising both from the measurement challenges of smaller feature sizes and the growing requirement to characterize structures in more than just a single critical dimension.^1,2^ These changing requirements have dramatically altered manufacturing metrology, both in terms of the available technology and its method of application.

In the area of scanning electron microscopy (SEM), there has been a shift from the basic edge operators used in the 1990s to increasingly sophisticated imaging models and the increasing use of tilt SEM to reconstruct three-dimensional (3-D) representations of the surface.^3,4^ The past two decades have also seen the prodigious rise of scatterometry or optical critical dimension (OCD) metrology for in-line process control.^5–7^ Despite steadily increasing demands on the accuracy of the required models, both SEM and scatterometry continue to be widely utilized for process metrology.

For reference metrology, critical dimension atomic force microscopes (CD-AFMs) remain well utilized, despite the demand for ever smaller, stiffer, and more robust tips. Furthermore, within the past decade, a new form of tilt AFM, with imaging capabilities similar to CD-AFM, has been introduced and is gaining acceptance in manufacturing support.^8,9^ An alternative implementation of CD-AFM based on the vector approach probing method has also been developed to enhance measuring flexibility as well as to reduce tip wear.^10^ Finally, the past decade has also witnessed the dramatic rise of interest in critical dimension small angle x-ray scattering (CDSAXS) as a potential reference metrology.^11,12^

Perhaps most dramatically, the realization that no single metrology technique will be suitable for all measurement goals has given rise to the concept of holistic and hybrid metrology, in which the results of multiple metrology methods are systematically combined so as to leverage the strengths of both and to lower the ultimate uncertainties.^13,14^

In general, the limitations of CD-AFM tend to be lower throughput relative to SEM and OCD, and it has image artifacts resulting from the tip sample geometrical and mechanical interaction. For reference metrology purposes, however, throughput is of secondary concern to the need for accuracy, and this, in turn, is largely dependent upon improvements in tip fabrication, characterization, and tip scanning control algorithms.

The persistent challenge for CD-AFM technology has been keeping pace with the need for smaller tips. When CD-AFM was initially developed, the ability to image a 0.5-µm trench was technologically relevant.^15^ The requirements for current technology are nearly a factor of 50 smaller. This has resulted in a steady evolution of CD-AFM tips toward smaller widths, but the need to maintain the lateral stiffness of tips has also meant that these tips had to be shorter. Smaller tips were even more susceptible to damage than larger ones, so there has also been development of nonsilicon CD tips, specifically those of high-density diamond-like carbon.^16^

Due to the stiffness and wear-resistance of carbon nanotubes (CNTs), there has also been an ongoing interest in the use of CNTs as AFM tips.^17–23^ Indeed, there are now some commercial vendors of CNT tips. However, there have been significant issues surrounding both manufacturing and using CNT tips. Specifically, the growth or attachment of CNTs to AFM tips and cantilevers has been a challenge to the fabrication of CNT tips, and the flexibility and resultant bending artifacts have presented challenges to using CNT tips.

CNT tips have several potential advantages over silicon-based tips: (1) significantly larger Young’s modulus by approximately a factor of five, (2) much greater wear resistance, and (3) a geometrical advantage over conical silicon tips in that wear of cylindrical CNT tip would not necessarily degrade its lateral resolution.^18^ Additionally, CNT tips will buckle under excessive compressive loading. This characteristic has the advantage of reducing sample damage, but brings with it the disadvantage of buckling-related imaging artifacts, as will be illustrated later.

Given the much greater prevalence of conventional AFM relative to CD-AFM, most of the research into CNT tips has involved conventional AFM. The ability to image near-vertical sidewalls is the central feature of CD-AFM, and this geometrical requirement means that the constraints on the CNT orientation for a useful tip are even greater than for conventional AFM tips. However, there has been progress in the application of CNT tips to CD-AFM by a number of researchers.^22,24^ The considerably greater wear resistance of CNT tips has also been demonstrated in this context.

In most cases, the goal of CNT tip fabrication has involved the use of straight CNTs. A cylindrical probe such as a vertical CNT can measure near-vertical sidewalls that are not re-entrant. However, for purposes of imaging vertical and re-entrant sidewalls, a tilted or bent CNT could be useful. Simply tilting a straight tip (or tilting the sample) carries a significant cost in terms of required geometrical clearance around a target feature. A vertical CNT tip with an intentional bend near the end offers the advantage of sidewall access but with much less stringent clearance requirements. Such a tip would allow access to sidewalls of one orientation. A more generally applicable solution would thus be a vertical CNT tip with lateral protrusions in both directions, thus more closely resembling the functional structure of silicon CD-AFM tips.

In conventional AFM imaging, two distinct and significant artifacts have been observed in the use of CNT tips: divots and ringing. These artifacts are thought to be related to CNT bending, adhesion, and stiction, and are affected by the operational parameters of the AFM.^20^ Generally, these effects are more problematic for longer and thinner CNTs. This is similarly the case for CD-AFM applications of CNT tips.

Approaches such as antiadhesion functionalization of the nanotube and the development of new AFM modes compatible with slender and flexible tips could also extend the duration and scope of CNT tip applicability. As we will show advances in CD-AFM scan algorithms for second- and third-generation instruments have increased compatibility with the CNT tips relative to first-generation tools.

An elegant example of using CNT tips to optimal advantage was demonstrated by Watanabe et al.^25^ These researchers leveraged the high lateral compliance of CNT tips by incorporating the bending behavior into a unique imaging mode that could measure vertical sidewalls with a straight and vertically mounted CNT tip. This innovative work helped set the stage for a more recent investigation of CNT snap-in behavior on vertical sidewalls using a second-generation CD-AFM.^26^ In the future, similarly creative ways of using the behavior of very small tips to advantage will likely become increasingly important at the frontier of CD-AFM metrology.

Since the early 2000s, the Korea Research Institute for Standards and Science (KRISS) has invested considerable effort in the controlled fabrication of CNT tips.^21,23^ Progress by KRISS in the precise control of CNT orientation, length, and end modification, using manipulation and focused ion beam processes, has allowed the implementation of ball-capped CNT tips and bent CNT tips for CD-AFM. Because the National Institute of Standards and Technology (NIST) has experience with CD-AFM metrology, the two organizations began collaborating during the mid-2000s on the application of CNT tips for CD-AFM metrology, and the initial results of this collaboration were published.^27^

In our initial effort, we used two different generations of CD-AFM instruments to evaluate ball-capped and bent-type CNT tips by imaging line/space gratings and a programmed line edge roughness specimen. We concluded that these CNT tips are capable of scanning the profiles of these features including re-entrant sidewalls, but there remain important challenges to address, including tighter control of tip geometry and careful optimization of scan parameters and algorithms for using CNT tips.

This paper describes those initial results in detail and the current status of the effort as well as our plans. The primary emphasis here is to identify and examine the artifacts that may occur in CD-AFM imaging using CNT tips. The remainder of this paper is organized as follows. In Sec. 2, we describe two types of CNT tips developed by KRISS. The fabrication of these probes is described in Sec. 3. In Sec. 4, we show the measurement results. Our conclusions are summarized in Sec. 5.

## 2 Types of Tips Developed by KRISS: Ball-Capped and Bent CNT Tips

**Fig. 1:**
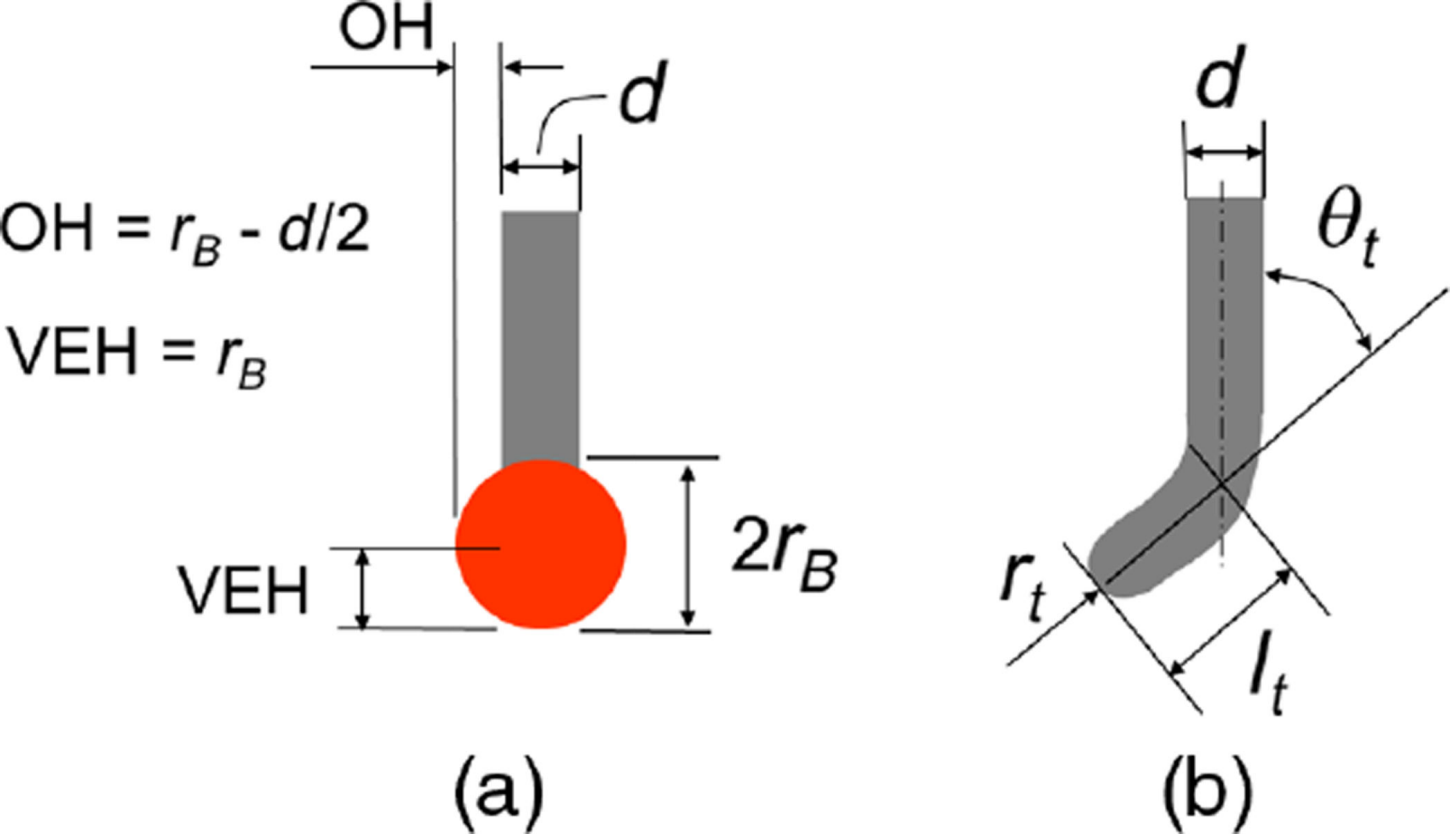
Geometry and tip characterization parameters for (a) ball-capped CNT tip and (b) bent CNT tip.

Figure 1 shows the types of CNT tips that we have developed at the Korea Research Institute of Standards and Science (KRISS) for new CD-probes. These are especially designed for dense lines where the tips should enter narrow trenches to probe the vertical sidewall surfaces. One is ball-capped (“B-tip” for short) and the other is bent (“J-tip” for short). The dimensions that we use to specify them are also illustrated.

It is worth observing that probes with these general shapes are useful in coordinate measuring machine (CMM) metrology and stylus profilometry at larger length scales. If such tips can be implemented for AFM, then metrologists will be able to enjoy similar probing capabilities at the nanometer scale.

The B-tip will provide similar advantages for AFM as the ball probe does for CMM metrology. It has a form that can enter the narrowest trenches or holes and probe both sidewalls. The extent of overhang or the degree of re-entrance that it can probe, however, is not as large as for the J-tip. The J-tip, on the other hand, is designed to probe one sidewall only, and cannot image the opposite one. It does, however, have a more effective geometry for imaging the bottom corner region—since the end is oriented at nearly normal to the local surface at the corner transition region—and in lithometrology applications, this region is of considerable interest. The J-tip provides a small vertical edge height (VEH), equal to the end radius of the CNT. This might be an advantage for some applications if the radius can be measured *a priori*. The J-tip may also be useful for experimental line-width measurement procedures in which two images taken in opposite sample orientation are stitched together to form a composite image.^28^

The functional geometry of CD-AFM tips is typically parameterized in terms of several metrics that help to quantify the expected performance and limitations of the tip for specific measurements. For our purposes here, we follow the most common convention, as described by Dahlen et al.^29^ The three metrics we will emphasize here are: (1) the tip width, which determines the smallest trench that can be measured, (2) the VEH, which limits the bottom portion of the sidewall that can be observed, and (3) the maximum overhang, which determines the limit of undercut or re-entrance of a sidewall that can actually be observed.

For purposes of comparison between silicon CD tips and our CNT tips, we can derive the same types of tip metrics from the design dimensions of the CNT tips. For the B-tip, the tip width is 2*r_B_*, the overhang is *r_B_* − *d*/2, and the VEH is *r_B_*, where *r_B_* is the radius of the ball and *d* is the diameter of the CNT shaft. For the J-tip, these dimensions are *l_t_* sin θ_*t*_ + *d*/2, *l_t_* sin θ_*t*_ − *d*/2, and *d*/2, respectively, where *l_t_* is the length of the bent portion of the CNT, θ_*t*_ is the angle of bending, and *d* is the diameter of the CNT shaft. For B-tips, a typical value of *d* is 40 nm, and a typical value of *r_B_* is 30 nm. For J-tips, a typical value of *l_t_* is 200 nm, and a typical value of θ_*t*_ is 30 deg.

## 3 Fabrication of Carbon Nanotube Tips at KRISS

**Fig. 2:**
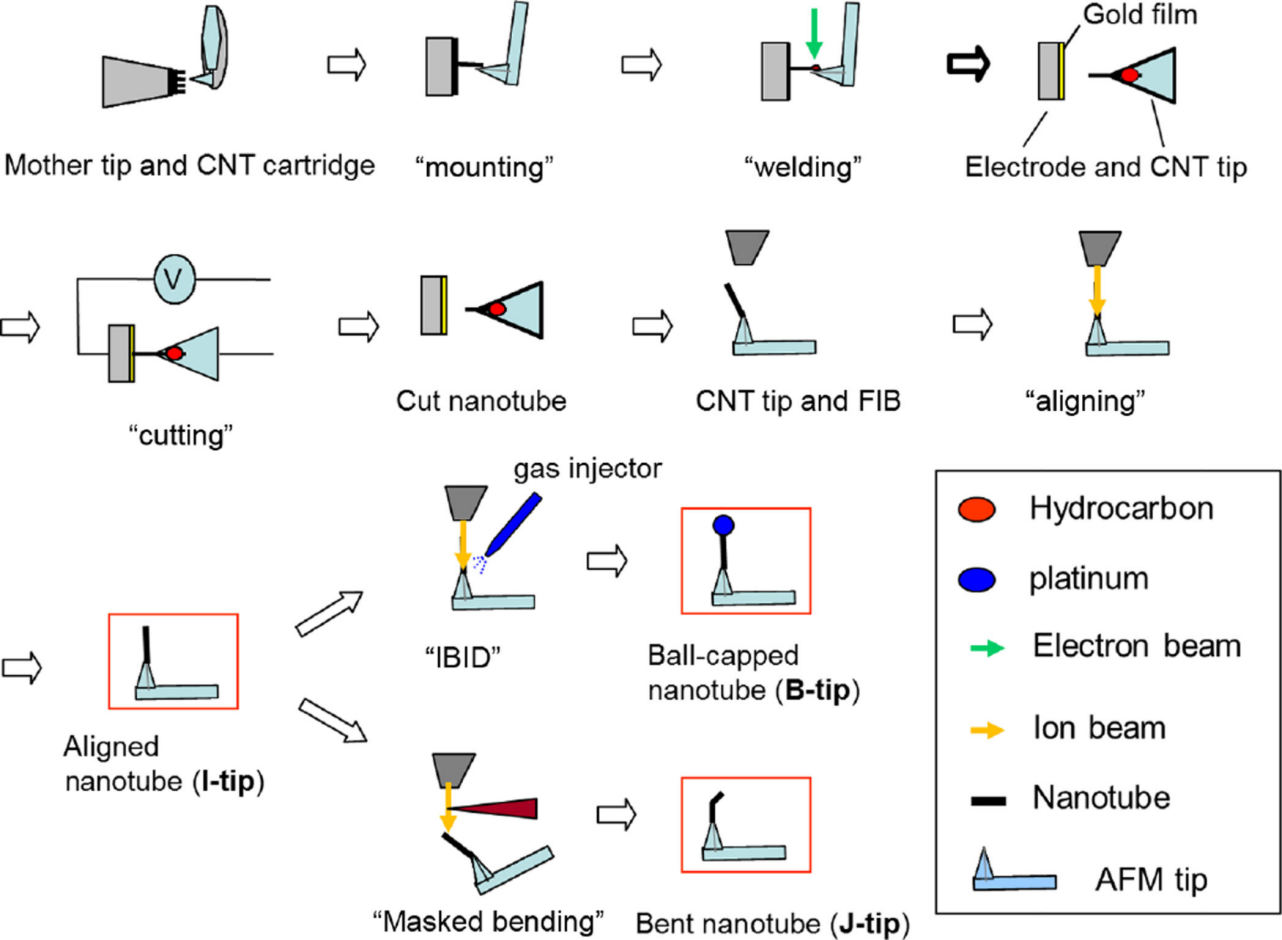
Schematic of the KRISS fabrication procedure for B-tips and J-tips.

It has been previously discovered that a focused ion beam (FIB) can align, bend, and straighten free-standing CNTs attached at one end to a substrate.^30^ Subsequently, it was discovered that sufficiently thin and high aspect ratio probes can be reproducibly treated the same way regardless of material composition.^31^ We also found that while the aligned CNTs were being irradiated by a parallel FIB from the top, a metal ball is formed at the CNT top end through ion beam-induced deposition (IBID). Since these processes are quite simple and are already described in the references, we do not discuss them in detail in this paper.^23^ By modifying the FIB process for straightening CNT tips on the manipulator, we have easily fabricated a B-tip as well as J-tips for CD-AFM as shown in Fig. 2.

At the present time, the arc discharge-grown technique provides the best-quality CNT for probes, with diameters ranging from 10 to 40 nm. In order to fabricate unique shapes for CD probes, the CNT is first attached to a base tip so that the protrusion can be well aligned and have suitable length.

There exist several methods reported so far for the fabrication of CNT tips. Each method has its own strengths and drawbacks. The simplest method is to attach a CNT directly to the base AFM tip manually using an acrylic adhesive while observing with an optical microscope.^17^ The method presents difficulties for accurate manipulation because an individual nanotube is not discernable with an optical microscope. A more sophisticated and expensive method is manufacturing in a vacuum chamber while viewing the individual nanotubes via a real time SEM image.^32^ Nanomanipulators installed in the SEM enable the manipulation with nanometer-level resolution. In contrast to the manipulation technique, some laboratories grow CNTs directly on AFM tips through chemical vapor deposition (CVD).^19^ With the CVD method, one can fabricate CNT tips on a wafer scale but some key issues not yet resolved include reproducible control of the length and growth direction of CNT. The FIB modification process described earlier can be applied to all CNT tips no matter how they are made, attached or directly grown, as long as they are not too poorly oriented.

Until now, the method of using nanomanipulation in an SEM chamber is considered to produce the most accurate CNT tips. The mounting of the CNT is performed with the manipulator installed in an SEM, where a CNT is placed on the rear surface of the pyramidal silicon AFM mother tip, and then is glued using electron beam induced deposition of hydrocarbon. While the manipulation has sufficient resolution in the horizontal plane, normal to the e-beam direction, the vertical position cannot be distinguished within 1 µm or so because of the large depth of field of the SEM image. This makes impossible the precise 3-D manipulation of the CNT and AFM tip in space, resulting in a wide distribution of CNT attachment angles. One can improve this method by enabling the rotational motion of either or both the AFM tip and the CNT, with respect to the axis of the AFM tip, achieving an angle alignment precision as small as about 10 deg.^33^

Our fabrication of CNT tips consists of two separate steps: mounting of the CNT onto the AFM mother tip using manipulation in an SEM and the subsequent CNT modification using FIB. The procedure is shown in Fig. 2. CNT tips fabricated by manipulation in SEM are usually misoriented as made, so they are aligned in the FIB system through the ion beam bending (IBB) process. Thus processed, a CNT tip acts as a normal straight CNT tip, and the CNT itself maintains its elasticity as can be checked with a bending test in SEM.^34^ Using this process, we can change a “bad” tip to a “good” tip and a “good” tip to an “optimized” tip. The subsequent modification of CNT tips for CD-AFM starts with this straight form. It is noteworthy, however, that although IBB improves the tip geometry, ion bombardment is believed to introduce defects in nanotube structures, so minimization of dose is recommended. It is equally important to have a good nanotube tip prior to the IBB process. IBB-aligned CNT tips are then bent further to make the J-tip by irradiating the CNT end not masked by the cantilever, since the exposed part aligns opposite to the FIB direction whereas the unexposed part remains as is. Alternatively, FIB aligned CNT tips may be formed into B-tips by depositing platinum at the end using IBID. The organic platinum molecule adsorbed on the CNT is dissociated, and the platinum atoms form a ball at the top end of the nanotube while minimizing the surface energy. As the ball increases in size, it masks the CNT below, like an umbrella, so that the total increase in the shaft thickness is not significant.

The optimum bending angle for a J-tip is dependent upon the desired application, since the geometry of the J-tip involves an inherent trade-off between performance on horizontal surfaces and vertical sidewalls. A bending angle of 90 deg might be optimum for imaging vertical sidewalls, since the tip would be normal to the surface. However, this would increase the geometrical clearance requirements of the tip in narrow trenches, and it would result in a blunt tip–sample contact region on horizontal surfaces, along with a corresponding impact on resolution.

In terms of tip fabrication, with the cantilever mask, we cannot simultaneously control the angle and length of the bent CNT, which requires the employment of an independent mask. There is, however, still some flexibility to control the bending angle by controlling the FIB irradiation time.

**Fig. 3:**
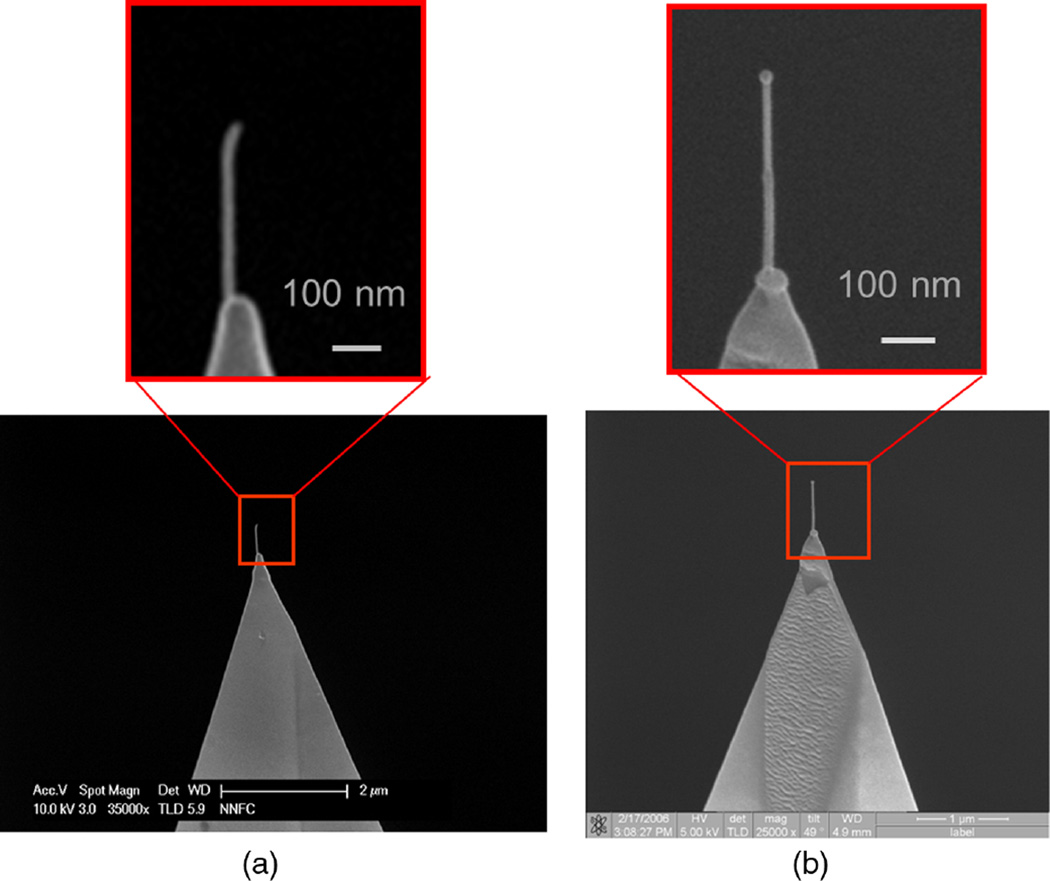
Fabricated nanoprobes for CD-AFM: (a) the J-tip and (b) the B-tip. These probes can scan the sidewalls of a feature.

Two kinds of tips, J-tips and B-tips, were made using the procedures described above. SEM images of J-tips and B-tips fabricated that were fabricated using these methods are shown in Fig. 3.

## 4 Measurements and Results

A complete description of CD-AFM principles and operation is outside the scope of this paper. However, the basic distinction between CD-AFM and conventional AFM is that CD-AFMs use two-axis (lateral and vertical) sensing of the tip–sample interaction and position control. In conventional AFM, the sensing of the tip–sample interaction is essentially limited to the vertical axis. When the two-axis tip control algorithm is combined with flared tips, it is possible to scan near-vertical and even re-entrant sidewalls.

NIST has been working with different implementations of CD-AFM technology since the early 2000s and has experience characterizing the performance and uncertainties of three different CD-AFM instruments. One of the major goals of this effort has been the application of CD-AFMs as reference measurement systems for traceable dimensional metrology.^35–37^ The measurements described in this paper were primarily obtained on two CD-AFMs: (1) CD-AFM1^35^ operates using a displacement measuring interferometer to detect the *z*-axis motion of the cantilever and (2) CD-AFM2^36^ operates using a variation on the optical lever method to sense the cantilever motion. CD-AFM1 is thus capable of operating with lower vibration amplitudes than CD-AFM2. However, the larger cantilever vibration of CD-AFM2 means that it is potentially more robust against tip sticking. A logistical side note is that CD-AFM2 is not owned by NIST but belongs to a collaborating organization and is installed at their facility. However, its performance and uncertainties were characterized by NIST, and NIST personnel were permitted access to the instrument at the time of these measurements. Subsequent to our initial efforts, some results were also obtained using CD-AFM3, a newer instrument that is installed at NIST.^37^ CD-AFM3 has a more advanced tip control algorithm for scanning which can potentially reduce some imaging artifacts of CNT tips.

In the following discussion, we elaborate on four experimental results that we have obtained by employing B- and J-tips in CD-AFM1 and CD-AFM2. For each measurement, the tip and sample geometries are given, and then the 3-D image and profiles are shown with short descriptions. The CNT tip-sample interaction is complicated and challenging to describe in general terms. More detailed models are being considered, but are beyond the scope of this paper.

**Fig. 4:**
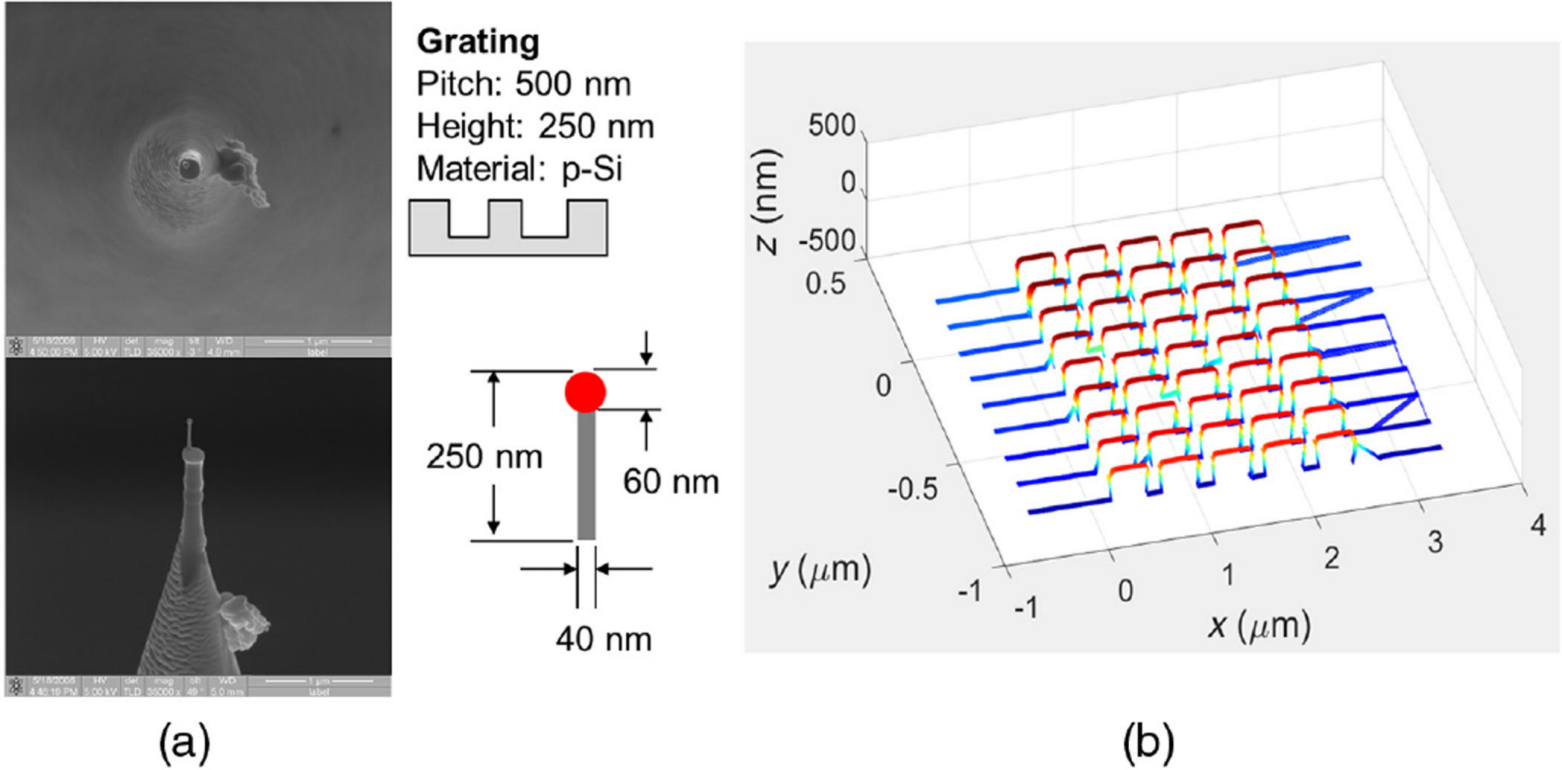
(a) The B-tip used and (b) the resulting CD-AFM1 image. In (a), the upper left is a top down SEM image of the tip; the lower left is a side view; and the right upper and lower sections show schematics of the grating and tip.

First, Fig. 4(a) shows the geometries of the platinum B-tip and the grating sample scanned in CD-AFM1. Figure 4(b) shows the resultant CD-AFM1 image composed of nine traces. The trace in front is for the tip moving to the right, the second trace is for the tip retracing and moving to the left, and so on. The measured profiles exhibit a slight re-entrance, consistent with the actual profiles, which indicates that the tip is functioning in concert with the two-dimensional feedback to track the actual sidewall. Due to the scale of the image, the sidewall re-entrance is not obvious, but it turns out to be ~10 nm, as observed both by silicon CD tips and CNT tips. More details on this sample are given in the Appendix, but our main goal here was the evaluation of CNT tip performance and not measurement of the grating. The length of each trace or scan line in Fig. 4(b) is nominally 4 µm, and each trace is offset by ~170 nm from the next one. The lines connecting the ends of the traces are not real but are just artifacts due to different numbers of adaptive sampling points in a trace and the plotting software used. This plotting artifact also appears in the rest of the CD-AFM1 and CD-AFM2 images in this paper.

**Fig. 5:**
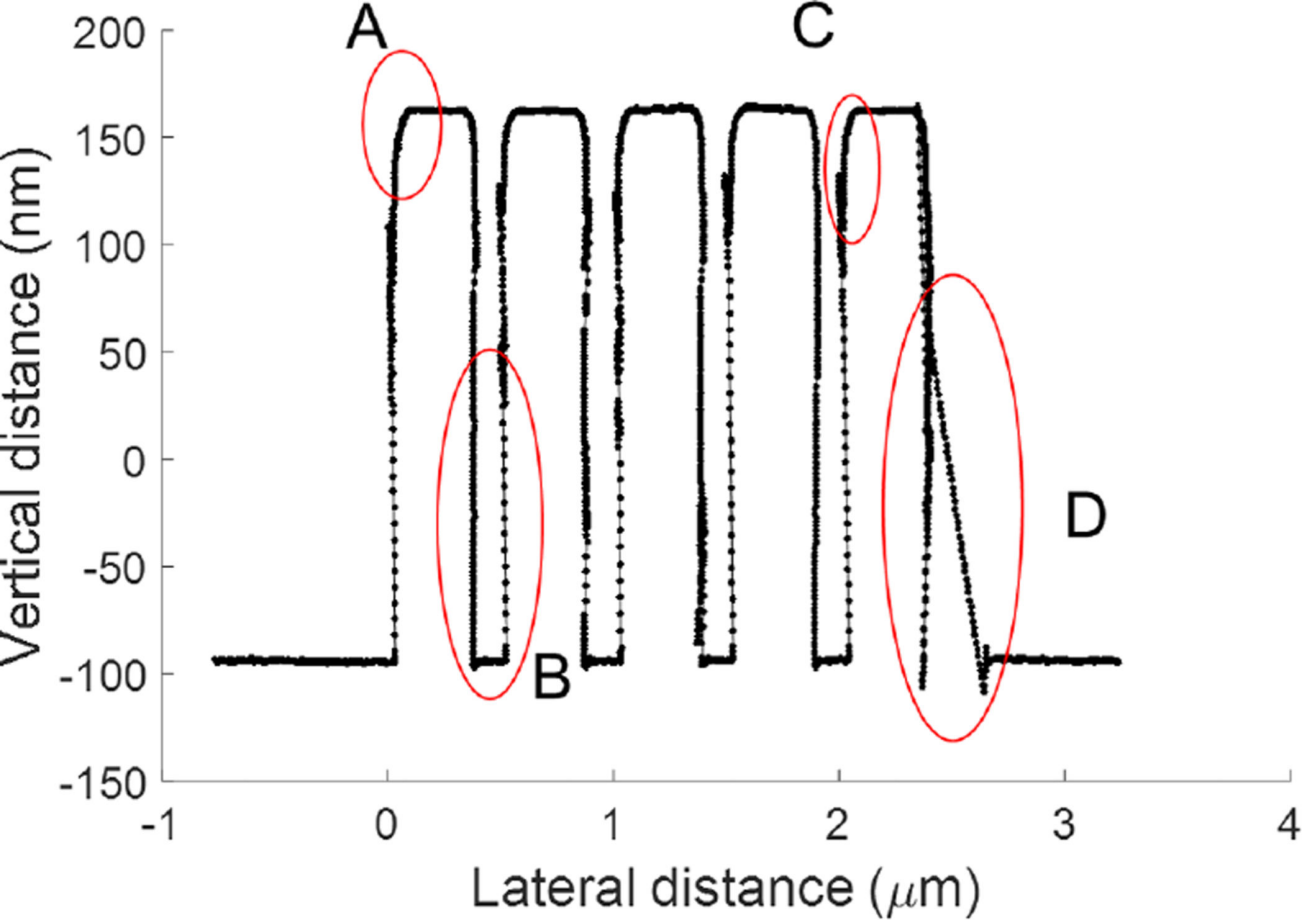
CD-AFM1 profile of grating sample obtained with a B-tip.

Figure 5 is the plot of one of the profiles from the image in Fig. 4(b). It has four typical artifacts found in CD-AFM profiles obtained with CNT tips. Artifact A in Fig. 5 is readily apparent. It is the rounding of the top corner, mostly or partly due to dilation from the interaction of the ball and the top corner. Artifact B is that the sampling interval is not uniform. This is because CD-AFMs operate using an adaptive scan mode, in which data are only taken when the surface sensing signal meets the condition set by the system. When the tip and sample are undergoing an abnormal or unstable interaction, such as that involved with flexing and sticking of the CNT tip, there will be significant portions of the profile for which there is no recorded data. In contrast, a typical CD-AFM flared tip shows more uniform sampling throughout most of the profile. Artifacts C and D are regarded as CNT tip-sample sticking either within or out of the range of modest control. The backtracking of the data as the tip scans up the sidewall (Artifact C) is extensively found in CD-AFM1 profiles, while artifact D is a rather typical distortion appearing in the profile when tip jumping occurs.

**Fig. 6:**
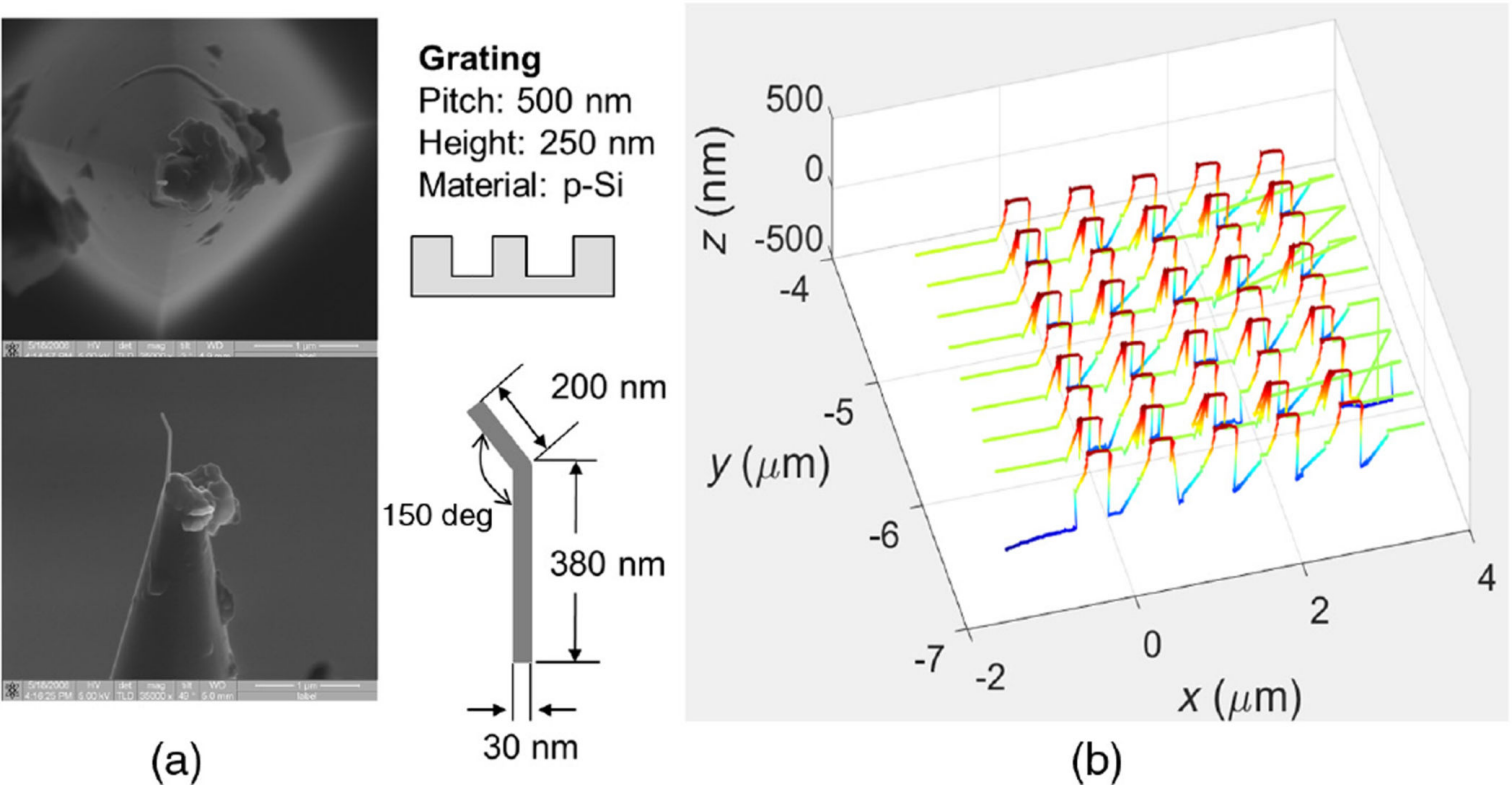
(a) The J-tip used and (b) the resulting CD-AFM1 image. In (a), the upper left is a top down SEM image of the tip; the lower left is a side view; and the right upper and lower sections show schematics of the grating and tip.

**Fig. 7:**
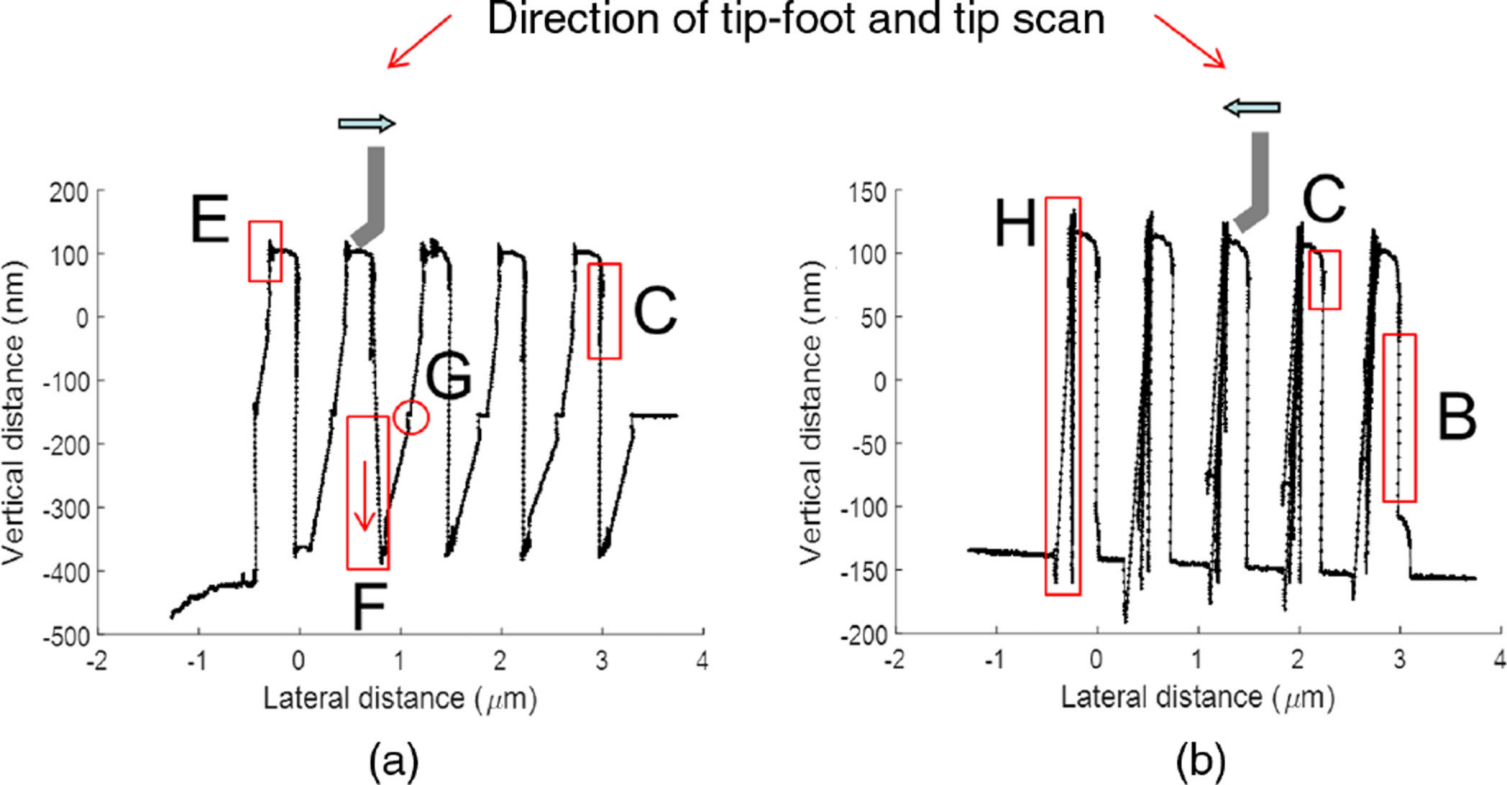
CD-AFM1 profiles of (a) trace and (b) retrace of a grating sample obtained with a J-tip, where six artifacts are indicated.

Figure 6(a) shows the geometries of the J-tip and the same grating sample as in Fig. 4. Figure 6(b) is the CD-AFM1 image obtained. The length of the trace is nominally 5 µm, and each trace is offset by ~250 nm from the next. The trace and retrace are quite different, each having different artifacts, indicating that the tip operation was abnormal. To look more closely at the artifacts, both the trace and retrace profiles are shown in Fig. 7.

Artifact E, as shown on the left of Fig. 7, is the result of feedback oscillation when the leftward foot of the J-tip is in contact with left tip corners of the grating. In normal operation, that part of the profile should have a rounded shape. More difficulty occurs in artifact F where the tip going down the sidewall begins to flex/buckle, and the cantilever keeps going, producing the apparent trenches (false height contrast), until the CNT relaxes to its undistorted shape at the point G. In the retrace, there are large feedback oscillations, artifact H, shown in the right of Fig. 7. These occur when the tip is scanning down the sidewalls, and it is expected that the vertical portion of the CNT is in contact with the sidewall as a whole. Additionally, the artifacts B and C that were observed using the B-tips (Fig. 5) are also apparent in the J-tip profiles shown in Fig. 7.

**Fig. 8:**
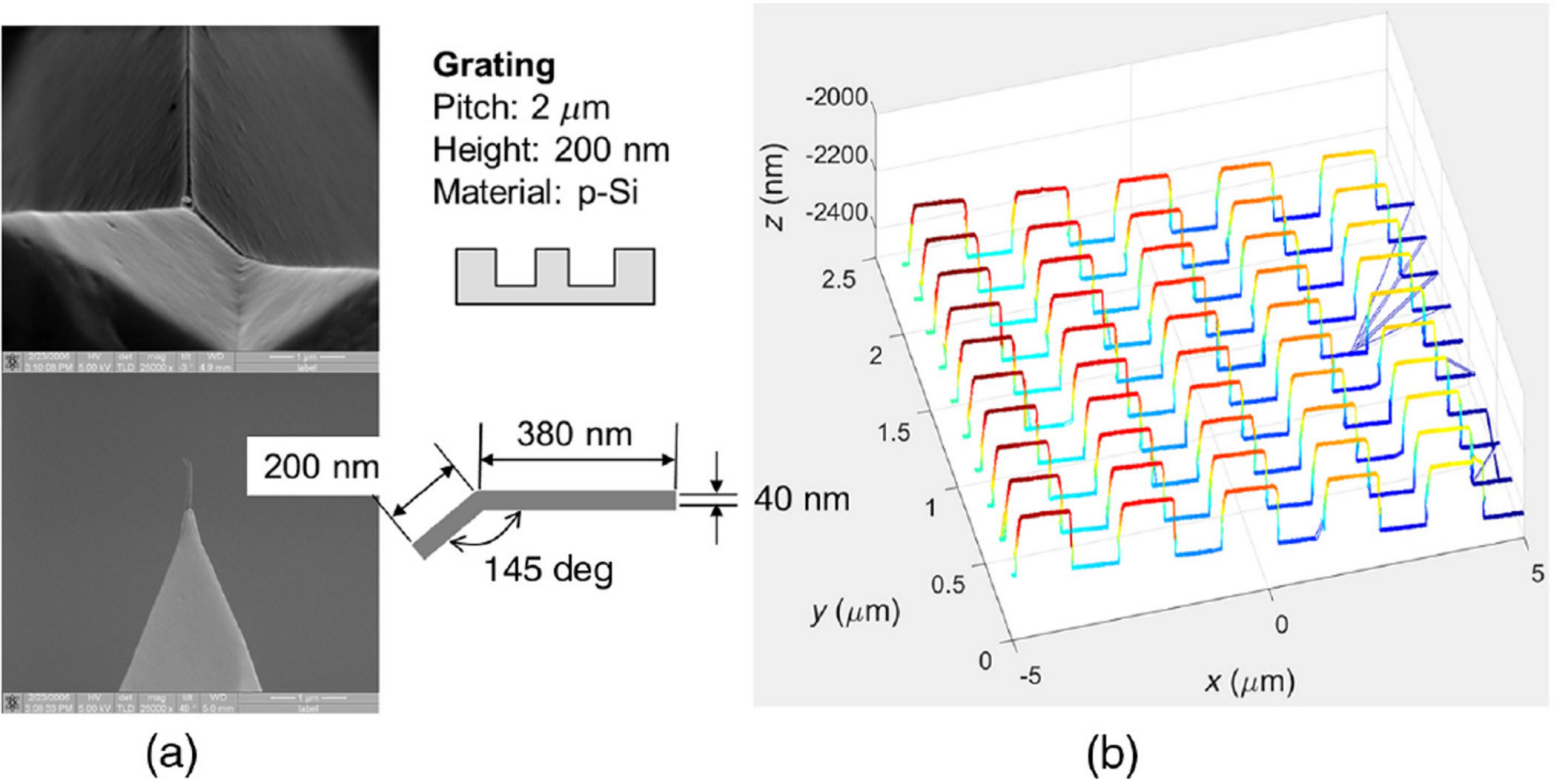
(a) The J-tip used and (b) the resulting CD-AFM2 image. In (a), the upper left is a top down SEM image of the tip; the lower left is a side view; and the right upper and lower sections show schematics of the grating and tip.

Those various profile artifacts either do not appear or are much smaller if standard flared silicon CD-AFM tips are used. This fact strongly suggests that the root cause is the slenderness/flexibility of the CNT, which results in tip flexing and tip-sample sticking during scans. These tip behaviors are not completely controlled by the AFM control algorithms, which were not originally developed for such flexible tips. CD-AFM2 has a more advanced tip control algorithm than CD-AFM1, so the performance of J-tips on CD-AFM2 was generally better than on CD-AFM1. However, since the instrument is located at a non-NIST facility, we used different tips and samples with CD-AFM2 than with CD-AFM1. Some of the results obtained using a J-tip with CD-AFM2 are shown in Fig. 8.

**Fig. 9:**
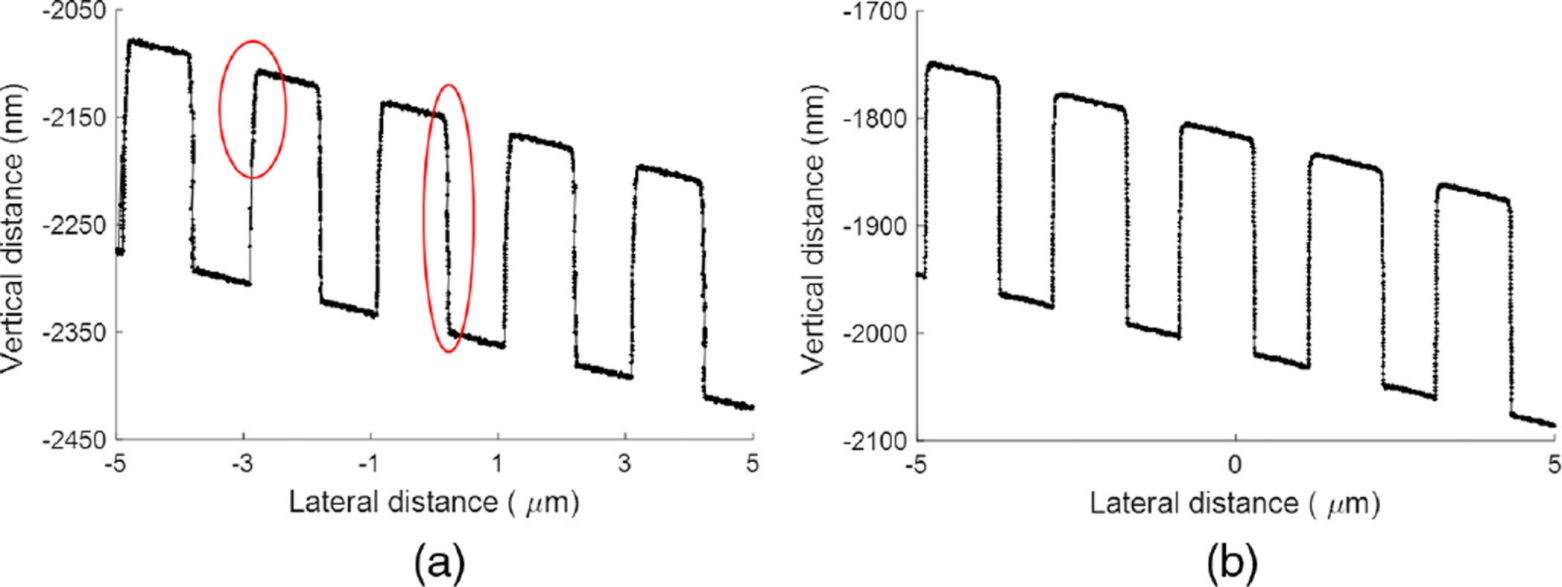
CD-AFM2 profiles obtained with (a) a J-tip and a stiffer I-tip, (b) a vertically oriented and shorter CNT tip. The sample scanned is a polysilicon grating with the pitch of 2000 nm and height of 200 nm.

Figure 8(a) shows the geometries of the J-tip and the grating sample. Figure 8(b) shows the image obtained with CD-AFM2 and is composed of five traces and five retraces, alternately. The length of the trace is 10 µm, and each trace is offset by 200 nm from the next one. At a glance we can see that the image is much cleaner than the earlier images. One of the profiles is shown in Fig. 9(a). The apparent sticking-related artifacts C and D are not seen anymore, except for nonuniform sampling intervals. This is because the abnormal signal is treated properly so that the empty points do not distort the measured profile. Assuming similar interactions of the J-tip and the grating, we can conclude that the scan algorithm for dealing with such interactions has superior performance in this instrument. For comparison, another CD-AFM2 image of the same grating but with the less flexible I-tip (i.e., a vertical and straight CNT tip) is shown in Fig. 9(b). Note that this grating has non-re-entrant sidewalls with an outward slope of about 85 deg. The sampling is quite uniform in the more densely spaced data points. Therefore, another possible conclusion is that the remaining issue of nonuniform sampling with the J-tip is still due to nanotube slenderness. This nonuniform sampling could be addressed by either stiffening the CNT or by more sophisticated control of the tip–sample interaction.

Apparent sidewall angles (SWA) were extracted from the measured profiles [Fig. 9(a), tip moving leftward] and were calculated using offline analysis software. The middle band of the sidewall (from 25% to 75% of the feature height) was determined for each edge and then the sidewall slope was determined from a linear fit to this segment of the edge. These results were averaged for all of the left and right edges in each scan line, and then averaged over all of the scan lines in each image. Table 1 shows the results of this SWA analysis for the J-tip scan shown in Fig. 9(a). The right edges of the features, which are in contact the end of tip foot, exhibit a larger apparent SWA than the left edges which contact the tip on the opposite side of the foot. Table 2 shows the apparent SWA extracted from a profile of the same grating imaged using an I-tip. The observed standard deviation of the SWA is relatively larger for the J-tip than for the I-tip, which means that the measurements using the J-tip were less stable. In contrast, the results obtained using the I-tip show symmetric SWA in the measured profile as well as better stability.

Another performance test we ran was to scan a programmed line edge roughness (LER) sample with CD-AFM2 using a J-tip. This type of LER sample was originally developed for testing CD-SEM measurements of LER during lithography development and optimization.^38^ The actual feature sidewalls are normally close to vertical, and may also be slightly re-entrant depending upon the details of the etch for a given sample. More discussion is included in the Appendix, but the primary characteristic of the programmed LER features is a periodic variation in linewidth having different spatial frequencies and amplitudes on different targets.

**Fig. 10:**
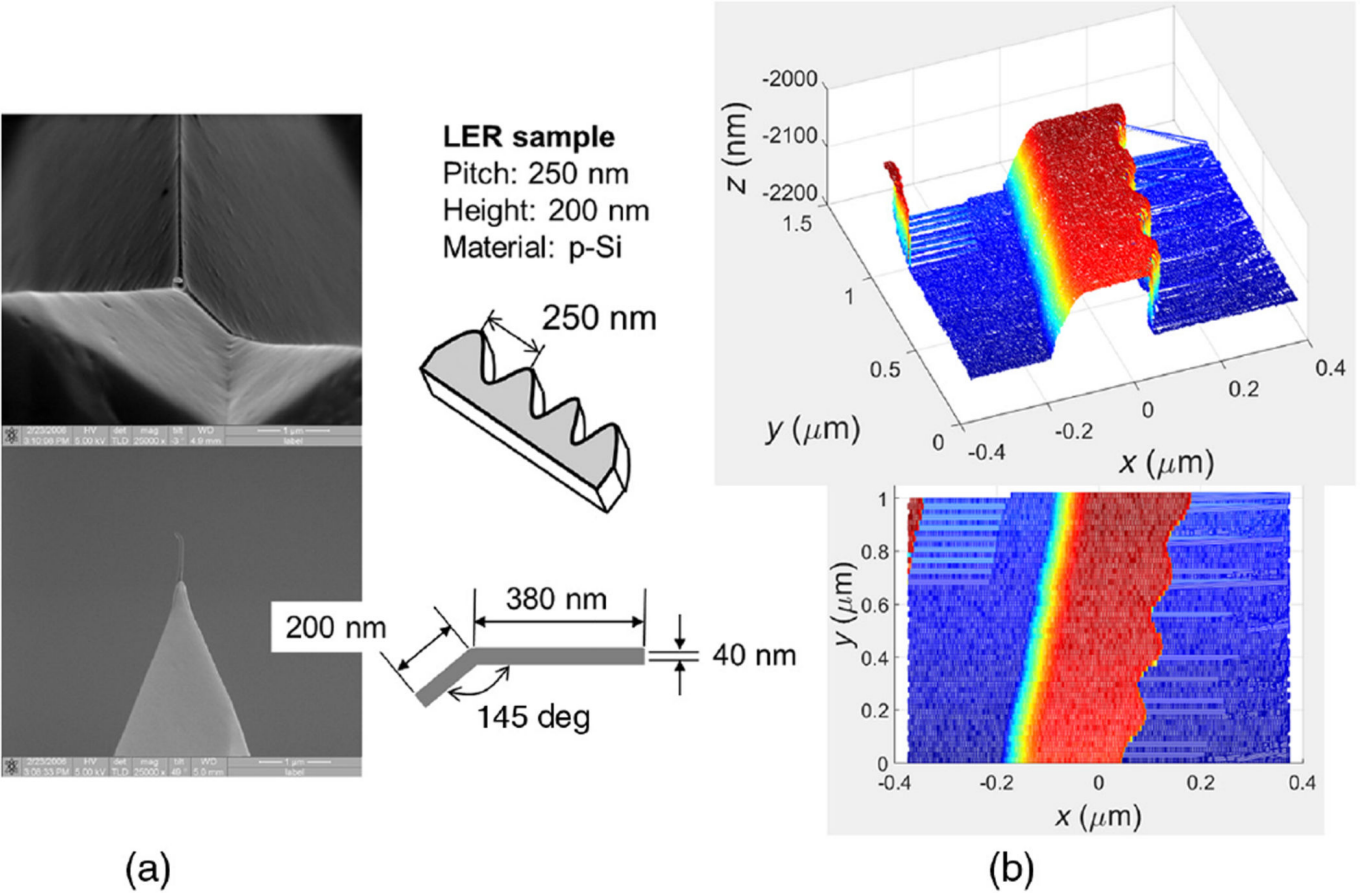
(a) The J-tip, same as in Fig. 8, and (b) the obtained CD-AFM2 image. In (a), the upper is a top-view SEM image of the tip and the lower a side-view image. The sample scanned was a polysilicon LER sample with the nominal spatial wavelength of LER and feature height of 250 and 100 nm, respectively. (Note that both renderings are of the same data, the upper one in 3-D rendering and lower in top-down.)

**Fig. 11:**
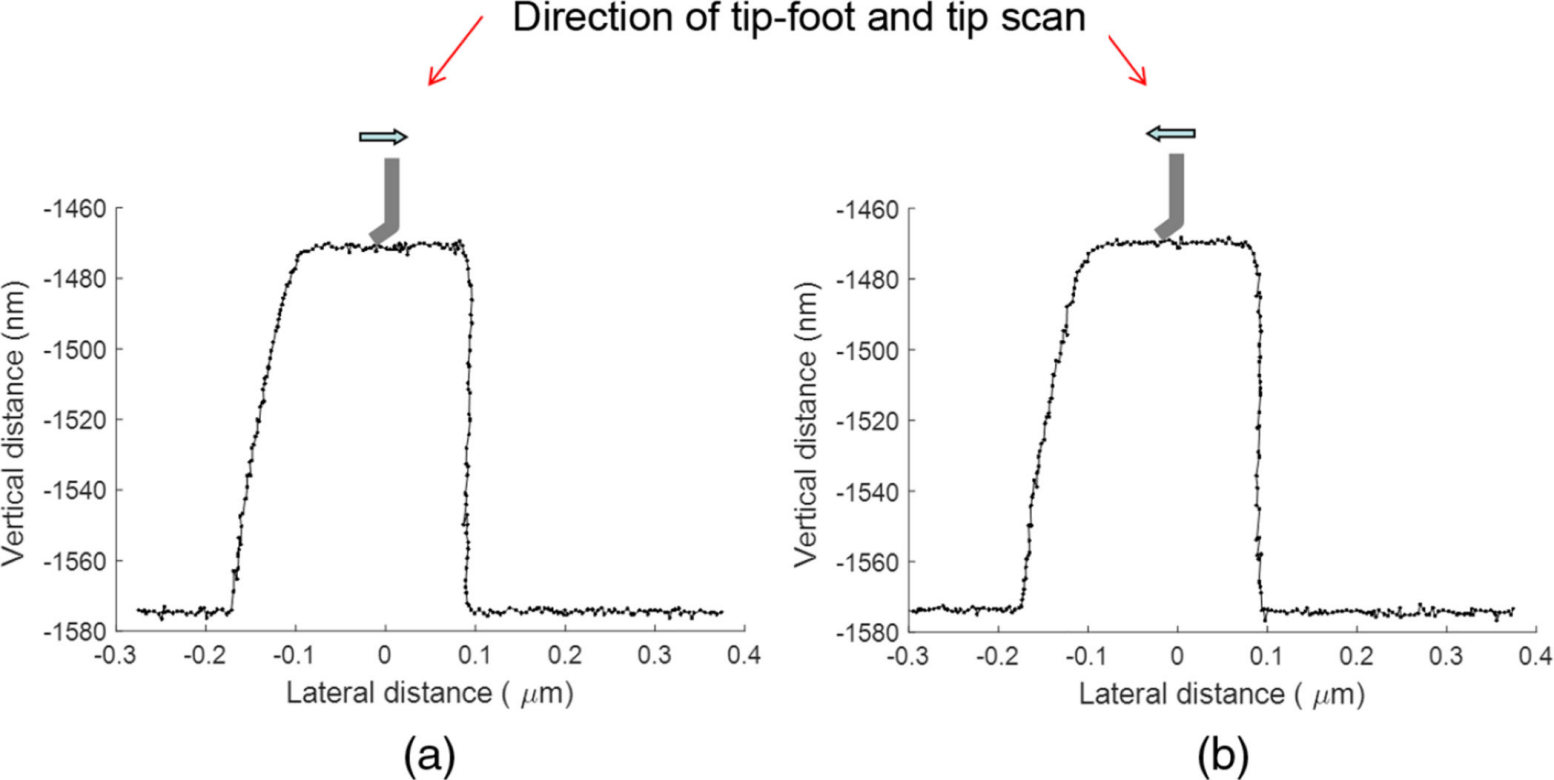
CD-AFM2 profiles of (a) trace and (b) retrace of LER sample obtained with a J-tip. The sample scanned was a polysilicon LER sample with the nominal spatial wavelength of LER and feature height of 250 and 100 nm, respectively.

A J-tip image of the LER sample taken using CD-AFM2 is shown in Fig. 10, along with the SEM images of the J-tip and schematics of the tip and sample geometries. The actual image is composed of 25 traces and retraces, and it is shown in both top-down and perspective views in Fig. 10(b). The length of each scan line is 10 µm, and each scan line is offset by 20 nm in the slow scan axis. The image shows that the performance of the tip during scanning was reasonably stable, and this is further seen in the extracted profile from the image shown in Fig. 11. The apparent rounding of the left edge is due to the tip–sample dilation. Since the leftward foot of the J-tip has a height which is comparable to the pattern height, most of the apparent left sidewall is a mirror image of the bent side of the J-tip. There is also no discernible difference between the trace and retrace profiles which was problematic for much of the CD-AFM1 data.

## 5 Summary and Conclusions

Using FIB processes that enable the geometrical modification of straight and aligned CNT tips, it is possible to fabricate CNT tips that are tailored for CD-AFM applications. Some examples developed by KRISS are the platinum ball-capped (B-tip) and the bent CNT tip (J-tip), which can reach re-entrant sidewalls and enter very narrow trenches or holes. The B-tip geometry more closely resembles the functional geometry of conventional CD-AFM tips and so is more generally applicable than the J-tip. However, as discussed more in the Appendix, the J-tip may be useful for cases of sidewall re-entrance exceeding 10 nm, which is the approximate limit of most conventional CD-AFM tips. Using two different instruments, KRISS and NIST evaluated the performance of these two tip types for CD-AFM metrology, and the initial results were promising.

Since our first experiments in this area, there have been improvements in CNT tip fabrication methods, both in general and specifically in terms of the performance of the system at KRISS. Implementing analog control of the nanomanipulation in the SEM has improved the accuracy of CNT mounting compared to the previous digital control system. Consequently, the subsequent step of FIB alignment is no longer necessary. We have also implemented a new method of CNT cutting that uses electron beam etching instead of electrical cutting. As a result, the cutting precision has been improved by about an order of magnitude: from 300 nm down to 30 nm.

There have also been improvements in CD-AFM technology since our initial work. Specifically, tip scanning and control algorithms show improved stability and performance on instruments such as CD-AFM3.We are currently conducting experiments on newer CNT tips using this instrument. A preliminary example of some of these results is included in the Appendix.

In our continuing work, we plan to test CNT tips of these types with dimensions optimized for more challenging samples, including different materials and narrower trenches. Using the flexible fabrication techniques described here, we also plan to develop new kinds of nanoprobes that can address CD and LER measurements for current technology nodes. Although our fabrication method is not compatible with the requirements of mass production, we believe that a demonstration of effective tip geometries will motivate the development of alternative wafer-scale production techniques by other laboratories and will enable the advancement of CD-AFM capability.

## Figures and Tables

**Fig. 12:**
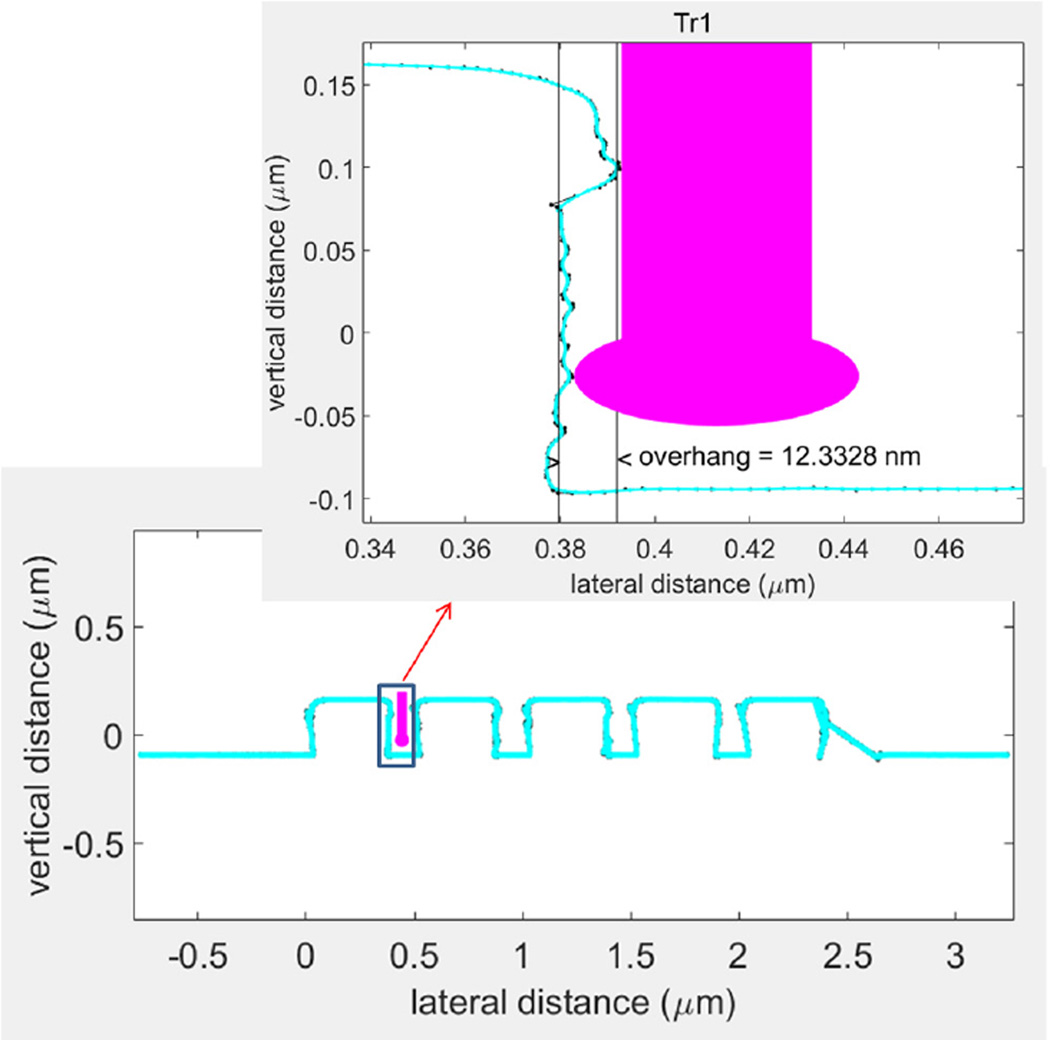
A 1:1 aspect ratio version of the grating profile from Fig. 5, along with an inset of a zoomed version on single edge.

**Fig. 13:**
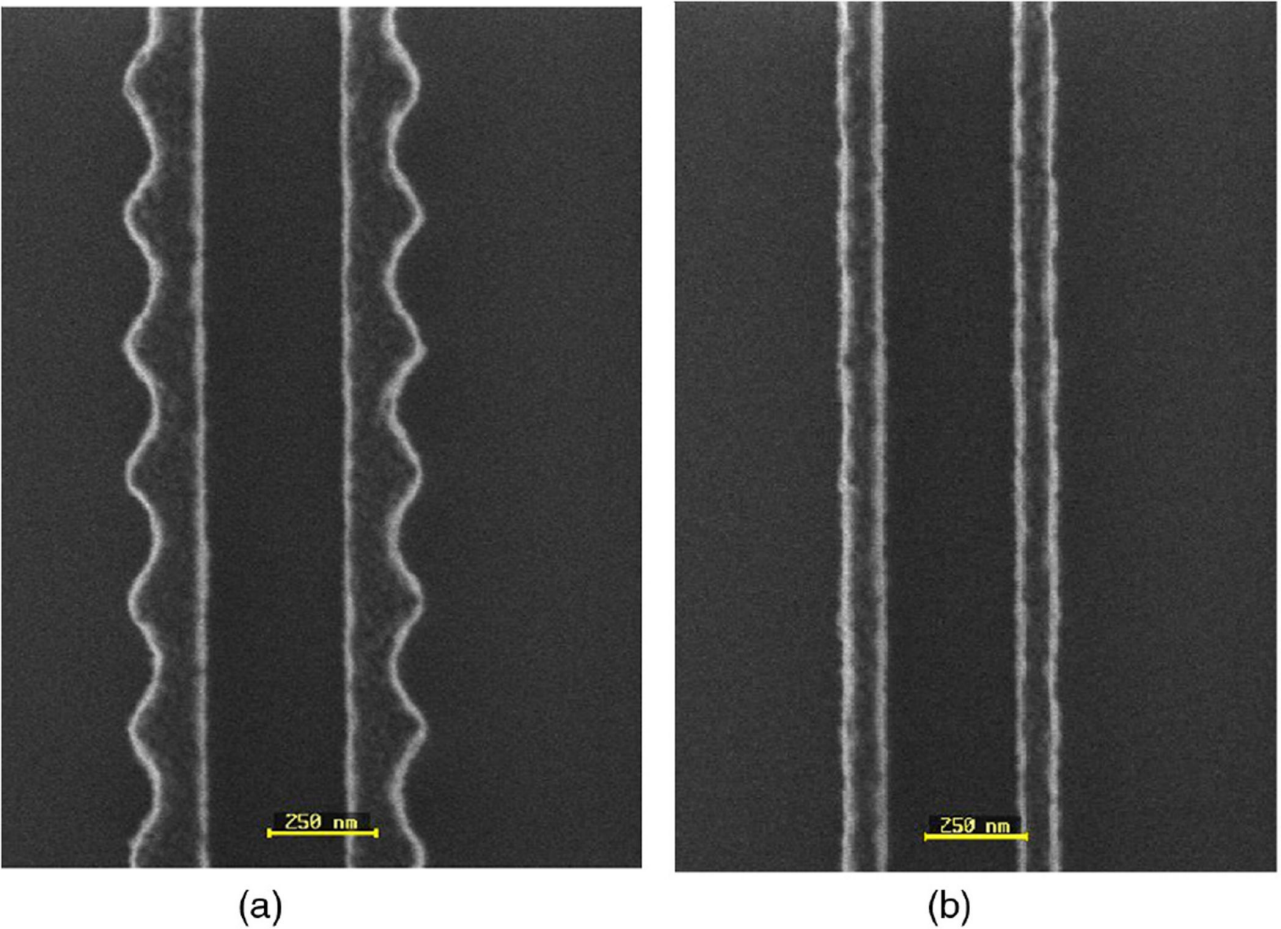
Top-down SEM images of programmed LER targets. These particular features had a spatial wavelength of 250 nm, but the amplitudes differed significantly due to differences in the lithography. The image in (a) corresponds with the sample we used in this work. The sample shown in image (b) had much lower amplitude and was not used for our work. (Images are courtesy of Ben Bunday, SUNYPoly and Andras Vladar, NIST.)

**Fig. 14:**
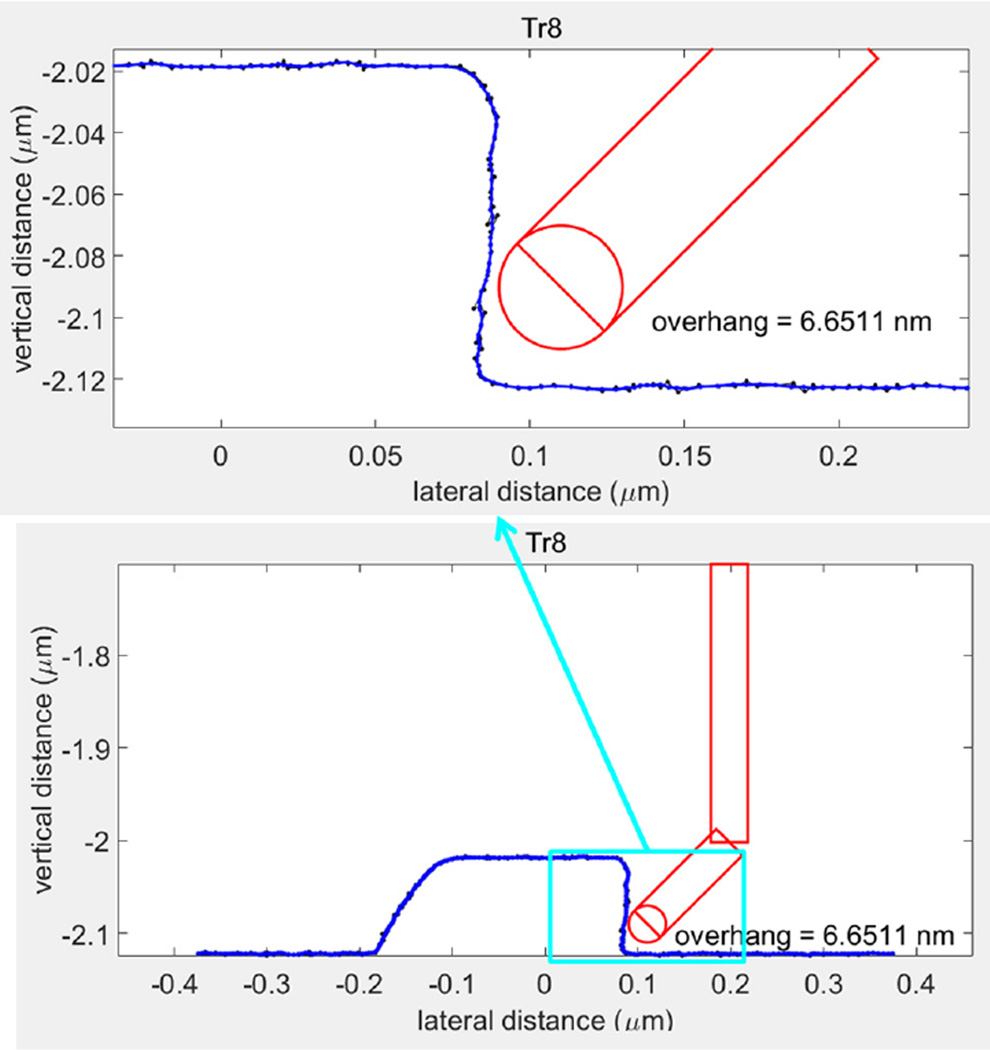
A 1:1 aspect ratio version of the LER structure from Fig. 10, along with an inset of a zoomed version on single edge that is accessible to the J-tip.

**Fig. 15:**
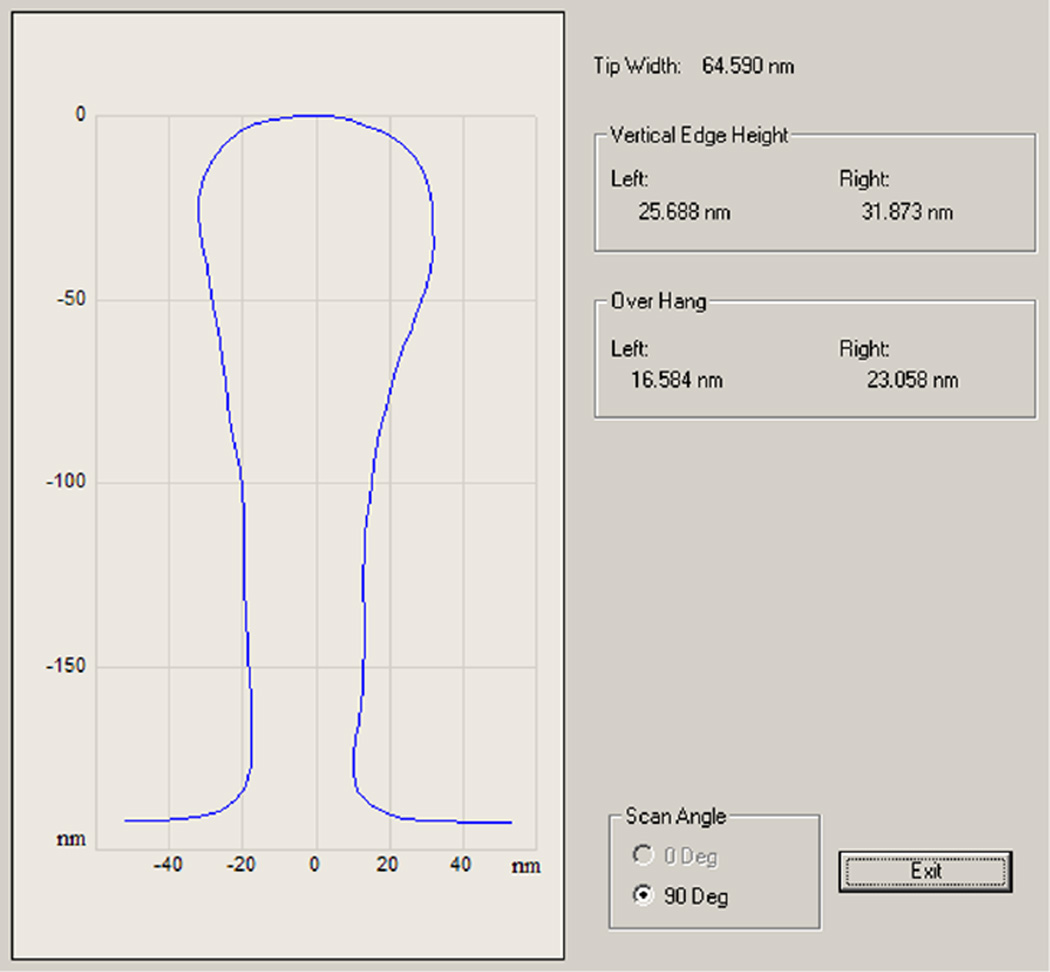
Output of routine tip qual procedure applied to an experimental B-tip recently tested on CD-AFM3.

**Table 1:** The apparent sidewall angles estimated from the profile of a grating sample measured with CD-AFM2 using a J-tip.

	Scan line number										Mean over scan lines	Standard deviation over scan lines

	1	2	3	4	5	6	7	8	9	10		
Left SWA (deg)	62.0	68.1	61.9	63.6	62.9	59.4	51.2	99.1	59.6	88.1	67.6	14.6
Right SWA (deg)	101.7	85.0	91.7	81.7	102.3	85.9	85.8	84.6	88.7	83.3	89.1	7.3

**Table 2:** The apparent sidewall angles estimated from the profile of a grating sample measured with CD-AFM2 using an I-tip.

	Scan line number										Mean over scan lines	Standard deviation over scan lines

	1	2	3	4	5	6	7	8	9	10		
Left SWA (deg)	82.5	83.2	83.7	84.2	83.2	83.1	82.4	83.1	83.0	82.9	83.1	0.5
Right SWA (deg)	83.6	83.5	84.0	83.6	83.6	84.3	84.0	83.9	83.5	83.7	83.8	0.3

## Appendix: Supplemental Measurements and Results

The line/space grating that was measured on CD-AFM1 using both a B-tip (Figs. 4 and 5) and J-tip (Figs. 6 and 7) has features that exhibit a slight re-entrance. These polysilicon lines have relatively large edge roughness and significant nonuniformity, but the amount of observed overhang on a feature sidewall is typically about 10 nm. While we did not obtain any TEM or SEM cross sections of these features, the observations of this overhang were comparable with both silicon and CNT tips.

The grating profile shown in Fig. 5 has a magnified *z*-axis scale relative to the lateral axis, which makes the re-entrance less obvious. In Fig. 12, two other versions of this profile are shown: (1) a 1:1 aspect ratio rendering of the profile and (2) a highly zoomed version on a single edge. Both of these are shown with a schematic representation of the tip shape. The observed overhang on the sidewall shown is ~12 nm. Using a conventional silicon CD tip to image the same target, we observed typical overhangs ranging between 8.5 and 12 nm. This means that with respect to overhang measurement on this grating, the performance of the CNT tip and silicon tips is equivalent.

The programmed LER sample used for the CD-AFM2 data shown in Figs. 10 and 11 was originally developed for evaluation of CD-SEM performance for LER metrology.^38^ As lithography continued to push toward smaller feature sizes, LER became an increasingly significant process issue, especially with the introduction of ArF resists. The spatial wavelength of this intentional LER varied from 50 to 500 nm, but the resulting amplitudes were significantly dependent upon the processing details, particularly the lithography. This is shown in Figs. 13(a) and 13(b) which are top-down SEM images of LER targets with a programmed spatial wavelength of 250 nm. But the details of the lithography performance resulted in significantly different amplitudes.

The geometry of the J-tip sacrifices access to one sidewall for improved access to the other. An example of a profile taken from the CD-AFM2 data on the LER sample, as shown in Fig. 10, is shown in Fig. 14, along with a scaled schematic of the J-tip geometry to illustrate the potential for measurement of re-entrant sidewalls. The actual overhang on this feature is only about 6 nm, but the tip geometry as shown would be capable of detecting more. Primarily, the performance would be limited by the slope of the re-entrant sidewall which should not exceed the bend angle of the J-tip. Provided that it does not and neglecting end radius effects, this particular tip would be capable, in principle, of detecting almost 100 nm of re-entrance in the configuration shown.

Finally, in Fig. 15, we show a preliminary result from recent experiments with newer CNT tips using CD-AFM3. This result was from a B-tip type fabricated at KRISS and evaluated at NIST. Figure 15 shows the output of the routine tip qual procedure: This involves scanning a known vertical sidewall structure to calibrate tip width and an undercut structure to characterize tip shape. The apparent tip shape shown is approximately as expected for a B-tip.

Currently, we are working to develop B-tips with widths less than 30 nm. However, with all CD-AFM tips there is the additional complication of the nongeometrical contributions to the effective tip width. Due to the lateral dithering motions involved in the control of CD-AFM tips, all CD-AFM tips exhibit an effective width that is larger than the purely geometrical width. Based on recent NIST studies in this area,^39,40^ we typically expect the magnitude of this contribution to be less than 10 nm, depending upon instrument settings and the control mode utilized. However, this subject remains an active area of investigation, especially for very small tips and new geometries, which may behave differently than expected.

We are continuing to test new CNT tips on CD-AFM3 to better understand the apparent tip qual results and with the larger goals of fabricating better tips and improving methods for using them. The possibility of developing tips with improved performance in very narrow trenches is one of our highest priorities.

## References

[1] Ukraintsev V, Banke B (2012). Review of reference metrology for nanotechnology: significance, challenges, and solutions. J. Micro/Nanolith. MEMS MOEMS.

[2] Vartanian V (2014). Metrology needs for through-silicon via fabrication. J. Micro/Nanolith. MEMS MOEMS.

[3] Kimura K (2003). Three-dimensional measurement by tilting & moving objective lens in CD-SEM. Proc. SPIE.

[4] Morokuma H (2004). New technique to reconstruct effective 3D profile from tilt images of CD-SEM. Proc. SPIE.

[5] Raymond CJ, Naqvi SSH, McNeil JR (1996). Scatterometry for CD measurements of etched structures. Proc. SPIE.

[6] Raymond CJ (2006). Dome scatterometry for the measurement of advanced geometry semiconductor devices. Proc. SPIE.

[7] Patrick H, Germer T (2007). Progress toward traceable nanoscale optical critical dimension metrology for semiconductors. Proc. SPIE.

[8] Cho S-J (2011). Three dimensional imaging of undercut and sidewall structures by atomic force microscopy. Rev. Sci. Instrum.

[9] Foucher J (2013). Introduction of next-generation 3D AFM for advanced process control. Proc. SPIE.

[10] Dai G (2012). New developments at Physikalisch Technische Bundesanstalt in three-dimensional atomic force microscopy with tapping and torsion atomic force microscopy mode and vector approach probing strategy. J. Micro/Nanolith. MEMS MOEMS.

[11] Settens C (2014). Assessment of critical dimension small-angle x-ray scattering measurement approaches for FinFET fabrication process monitoring. J. Micro/Nanolith. MEMS MOEMS.

[12] Sunday DF (2016). Evaluation of the effect of data quality on the profile uncertainty of critical dimension small angle x-ray scattering. J. Micro/Nanolith. MEMS MOEMS.

[13] Vaid A (2014). Hybrid metrology co-optimization of critical dimension scanning electron microscope and optical critical dimension. J. Micro/Nanolith. MEMS MOEMS.

[14] Henn MA (2015). Optimizing hybrid metrology: rigorous implementation of Bayesian and combined regression. J. Micro/Nanolith. MEMS MOEMS.

[15] Martin Y, Wickramasinghe HK (1994). Method for imaging sidewalls by atomic force microscopy. Appl. Phys. Lett.

[16] Foucher J (2012). Overcoming silicon limitations: new 3D-AFM carbon tips with constantly high resolution for sub-28 nm node semiconductor requirements. Proc. SPIE.

[17] Dai H (1996). Nanotubes as nanoprobes in scanning probe microscopy. Nature.

[18] Nguyen CV (2001). Carbon nanotube tip probes: stability and lateral resolution in scanning probe microscopy and application to surface science in semiconductors. Nanotechnology.

[19] Nguyen CV (2002). Carbon nanotube scanning probe for surface profiling of DUV and 193 nm photoresist pattern. Proc. SPIE.

[20] Straus MC (2005). Imaging artifacts in atomic force microscopy with carbon nanotube tips. Nanotechnology.

[21] Park BC (2005). Precision nanotube tip for critical dimension measurement with atomic force microscope. Proc. SPIE.

[22] Liu H-C (2006). Carbon nanotube AFM probes for microlithography process control. Proc. SPIE.

[23] Park BC (2006). Carbon nanotube probes for three dimensional critical dimension metrology. Proc. SPIE.

[24] Murayama K (2006). Three-dimensional metrology with side-wall measurement using tilt-scanning operation in digital probing AFM. Proc. SPIE.

[25] Watanabe M (2012). Atomic force microscope method for sidewall measurement through carbon nanotube deformation correction. J. Micro/Nanolith. MEMS MOEMS.

[26] Ukraintsev VA (2013). Distributed force probe bending model of CD-AFM bias. J. Micro/Nanolith. MEMS MOEMS.

[27] Park BC (2007). Applications of carbon nanotube probes in a critical dimension atomic force microscope. Proc. SPIE.

[28] Zhao X (2004). An image stitching method to eliminate the distortion of the sidewall in linewidth measurement. Proc. SPIE.

[29] Dahlen G (2005). Tip characterization and surface reconstruction of complex structures with critical dimension. J. Vac. Sci. Technol. B.

[30] Park BC (2006). Bending of a carbon nanotube in vacuum using a focused ion beam. Adv. Mater.

[31] Park BC (2006). Ion beam bending of nano scale materials in free space. Jpn. J. Appl. Phys.

[32] Nishijima H (1999). Carbon-nanotube tips for scanning probe microscopy: preparation by a controlled process and observation of deoxyribonucleic acid. Appl. Phys. Lett.

[33] Song WY (2005). Accuracy improvement of protrusion angle of carbon nanotube tips by precision multiaxis nanomanipulator. Rev. Sci. Instrum.

[34] Park BC (2005). Precision nanotube tip for critical dimension measurement with atomic force microscope. Proc. SPIE.

[35] Kramar JA, Dixson R, Orji NG (2011). Scanning probe microscope dimensional metrology at NIST. Meas. Sci. Technol.

[36] Orji NG (2007). Progress on implementation of a reference measurement system based on a critical-dimension atomic force microscope. J. Micro/Nanolith. MEMS MOEMS.

[37] Dixson R (2012). Traceable calibration of a critical dimension atomic force microscope. J. Micro/Nanolith. MEMS MOEMS.

[38] Bunday BD (2003). CD-SEM measurement line-edge roughness test patterns for 193-nm lithography. Proc. SPIE.

[39] Dixson R, Ng BP, Orji NG (2014). Effects of lateral tip control in CD-AFM width metrology. Meas. Sci. Technol.

[40] Dixson R (2015). Interaction of higher order tip effects in CD-AFM width metrology. J. Vac. Sci. Technol. B.

